# Phylogeography of the Patagonian otter *Lontra provocax*: adaptive divergence to marine habitat or signature of southern glacial refugia?

**DOI:** 10.1186/1471-2148-11-53

**Published:** 2011-02-28

**Authors:** Juliana A Vianna, Gonzalo Medina-Vogel, Claudio Chehébar, Walter Sielfeld, Carlos Olavarría, Sylvain Faugeron

**Affiliations:** 1 Departamento de Ecología y Biodiversidad, Facultad de Ecología y Recursos Naturales, Universidad Andrés Bello, Republica 440, Santiago, Chile; 2 Center for Advanced Studies in Ecology and Biodiversity, Facultad de Ciencias Biológicas, Pontificia Universidad Católica de Chile, Alameda 340, Santiago, Chile; 3 Departamento de Ecosistemas y Medio Ambiente, Facultad de Agronomía e Ingeniería Forestal, Pontificia Universidad Católica de Chile, Vicuña MacKenna 4860, Santiago, Chile; 4 Delegación Regional Patagonia, Administración de Parques Nacionales, Vice Almirante O'Connor 1188, 8400, San Carlos de Bariloche, Argentina; 5 Universidad Arturo Prat, Departamento de Ciencias del Mar, Av. Arturo Prat 2120, Iquique, Chile; 6 Fundación CEQUA, Plaza Muñoz Gamero 1055, Punta Arenas, Chile

## Abstract

**Background:**

A number of studies have described the extension of ice cover in western Patagonia during the Last Glacial Maximum, providing evidence of a complete cover of terrestrial habitat from 41°S to 56°S and two main refugia, one in south-eastern Tierra del Fuego and the other north of the Chiloé Island. However, recent evidence of high genetic diversity in Patagonian river species suggests the existence of aquatic refugia in this region. Here, we further test this hypothesis based on phylogeographic inferences from a semi-aquatic species that is a top predator of river and marine fauna, the huillín or Southern river otter (*Lontra provocax*).

**Results:**

We examined mtDNA sequences of the control region, ND5 and Cytochrome-b (2151 bp in total) in 75 samples of *L. provocax *from 21 locations in river and marine habitats. Phylogenetic analysis illustrates two main divergent clades for *L. provocax *in continental freshwater habitat. A highly diverse clade was represented by haplotypes from the marine habitat of the Southern Fjords and Channels (SFC) region (43°38' to 53°08'S), whereas only one of these haplotypes was paraphyletic and associated with northern river haplotypes.

**Conclusions:**

Our data support the hypothesis of the persistence of *L. provocax *in western Patagonia, south of the ice sheet limit, during last glacial maximum (41°S latitude). This limit also corresponds to a strong environmental change, which might have spurred *L. provocax *differentiation between the two environments.

## Background

**Figure 1:**
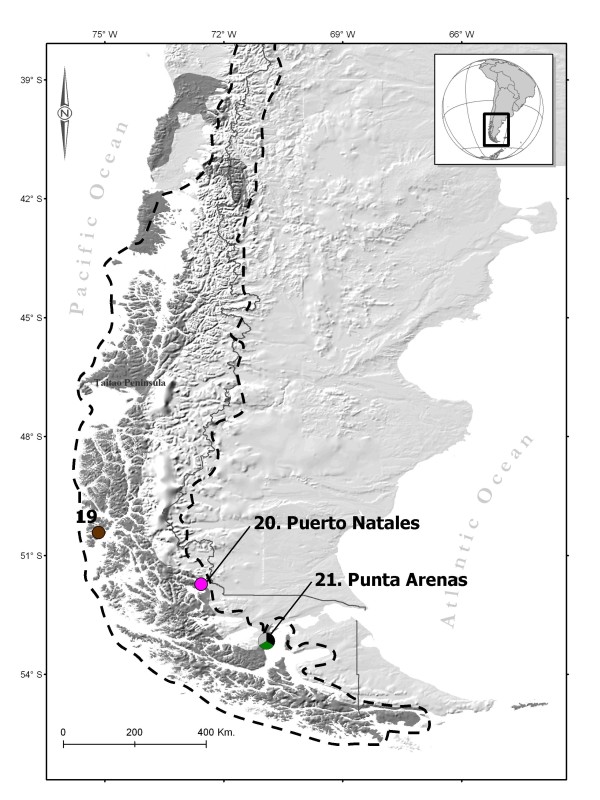
**Map of *Lontra provocax *distribution**. *Lontra provocax *distribution in dark gray (Medina, 1996) and ice sheet coverage limit during the Last Glacial Maximum in dashed line (McCulloch et al., 2000), including the three southernmost sampling sites (19, 20, 21 see Table 1). The haplotypes are represented by different colours such as the figure 4.

Climate change caused substantial alterations of the landscape and sea level, influencing patterns of species distribution. Pleistocene glaciations, ice-sheet advances and retreats in western Patagonia shaped land fragmentation, and the formation of islands and fjords along the Pacific Coast [1]. Throughout the Last Glacial Maximum (LGM,~25 000-15 000 years ago), ice sheets extended from 56°S up to 35°S along the Andes mountain range, and to 41°S in lowland areas and at sea-level in South America [2,3]. The southern fjords and channels (SFC) of southern Chile (41°S to 56°S, Figure 1) were covered by an extensive ice sheet. The pollen record indicates major shifts in most species of plants in this region [4]. As a result of the expected pattern of species range contractions and subsequent expansion after postglacial ice sheet retreat, genetic signatures of low diversity and demographic expansions are expected in these newly colonized areas [5,6]. This is the case for a vast majority of sigmodontine rodents, either from lowlands, Patagonian mountains or Tierra de Fuego [7-9]. Other studies, however, have suggested the persistence of freshwater species in the region throughout the last glacial period [10-13]. In the case of the Patagonian freshwater fish *Galaxias platei*, distributed along the western side of the Andes (39°S - 49°S), survival could have occurred in a southern refuge, possibly due to discontinuities of the ice field [12]. Likewise, southern refugia of the freshwater crab *Aegla alacalufi *has been suggested at the El Amarillo hot springs, which was possibly left uncovered by glacial ice, but isolated from more northern refugia [13]. Thus, it seems populations of freshwater species were not completely extirpated by ice cover in southern areas. This alternative pattern addresses important questions about the history of this region, the magnitude of the ice cover extension, and the consequences for the phylogeographic pattern of species associated with the SFC system for which there is a complete absence of data.

**Figure 2:**
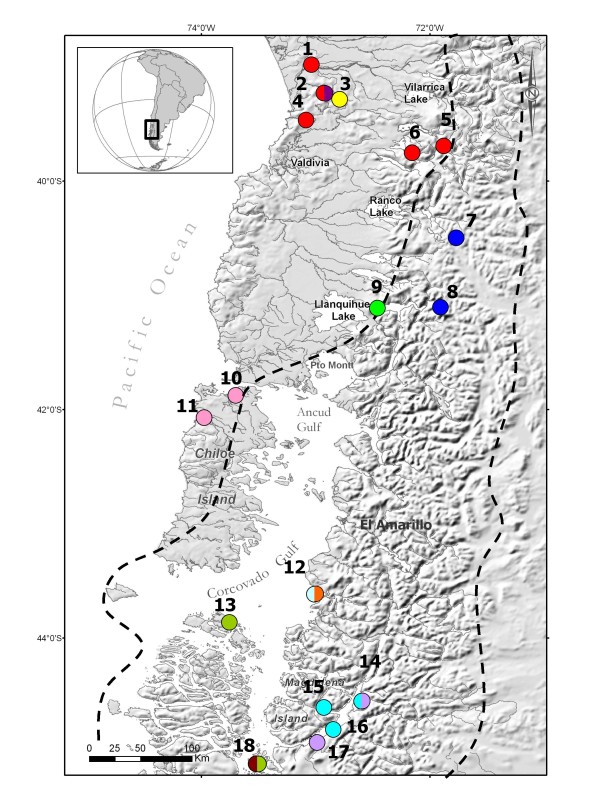
**Map of *Lontra provocax *samples collected in the northern part of the study area (1 to 18 according to Table 1)**. Ice sheet coverage limit during the Last Glacial Maximum in dashed line (McCulloch et al., 2000). The haplotypes are represented by different colours such as the figure 4. Petrohue River is respresented by the location 9.

In Chile, the northern glacial limit is also a boundary for major environmental changes (e.g. topography, currents, water salinity), resulting in a major biogeographic transition for marine [14] and freshwater species [15]. This biogeographic boundary is marked by the oceanic divergence between the Humboldt and the Cape Horn current systems, contributing to the relative isolation of the SFC marine fauna [16,17]. The SFC ecosystem is supplied by marine currents such as those from subantarctic waters. These become mixed with freshwater from abundant precipitation, river flow, and glacial meltwater [18]. Across the biogeographic boundary, hydrographic conditions change in freshwater environments, based on the weather, slope and lithology [19]. Two provinces are described for the biogeography of the freshwater fishes: the Chilean province, with a southern area of endemism from Valdivia River to Chiloé Island, and the Patagonian province, which is restricted to the western watersheds of continental Chiloé south to Tierra del Fuego [15]. Different scenarios altered the species assemblages across biogeographical limits and likely generated distinct patterns of genetic diversity. Phylogeographic inferences should help to understand species evolutionary history across marine and freshwater biogeographic breaks. Here we use molecular markers to address the hypothesis of glacial refugia on an aquatic top predator, the Huillín or Southern river otter, *Lontra provocax*. *L. provocax *populations extend across the biogeographic boundary, from exclusively freshwater habitat north of this limit to mostly marine habitat in the SFC (Figure 1, 2).

*L. provocax *has the smallest geographical range of all otter species [20], being distributed across the Andean-Patagonian region of southern Chile and part of Argentina [21-23]. In continental waters, the species is found from the Toltén River basin (39°S latitude) to the south of Chile [21]. The range extends through a limited area of the Andes mountains into Nahuel Huapi National Park and Limay River in Argentina [24-26]. South of Chiloé Island in Chile (42°S) the species also occurs in marine habitats along the SFC south to 56°S [27]. In the SFC south of Taitao Peninsula (46°S latitude), however, its distribution becomes exclusively marine [28,27]. As *L. provocax *is highly dependent on the availability of crustacean prey [29,30], the absence of the species from continental waters is related to the absence of crustaceans in the oligotrophic waters. *L. provocax *is solitary with intrasexual territoriality and an average home range of 11.3 km in rivers [31]. Dispersal is limited, as shown by radio-tracked otters, where the only long-range movement was made by a juvenile male that migrated 46 km downstream after release [31]. In freshwater, occurrence of *L. provocax *is dependent on crustacean distribution, which is strongly influenced by the river slope and altitude [32]. Consequently, the distribution of *L. provocax *along rivers is mostly concentrated below 300 m altitude [33], which may limit gene flow across the Andes mountain range. As crustacean and freshwater fishes were able to live in southern Chile during the LGM, *L. provocax *could have had a sufficient amount of food to survive during this period. However, the occurrence of *L. provocax *is also dependent on riparian vegetation. Terrestrial plants such as *Fitzroya cupressoides *and *Hypochaeris palustris *were mostly absent along ice sheet cover areas during LGM [34,35]. Thus, the question of *L. provocax *persistence during LGM on southern Chile likely implies a trade off between availability of food and terrestrial habitat.

So far the species has been studied mainly in the rivers and lakes at the northern limit of its distribution. Comparisons between specimens living in freshwater and marine environment are restricted to diet, and consists mainly of crustaceans such as *Samastacus *sp. and *Aegla *sp. in rivers and lakes [24,29], shifting to marine fishes of the genus *Patagonotothen *sp., crustaceans [36] and sea urchins in the marine environment. Although otter species occur mostly in freshwater habitats, the majority of these species have also been recorded in coastal environments [30]. Otters distributed along the coast, however, also need access to fresh water for drinking and washing their dense fur to remove accumulating salt and maintain thermo-insulation [30]. Fresh water is abundant along the SFC and *L. provocax *has also been recorded in inland rivers, such as Queulat River. It is important to note that, among all otter species, only two are exclusively adapted to marine habitats, i.e. the north Pacific Ocean sea otter (*Enhydra lutris*) and the chungungo or south Pacific marine otter (*Lontra felina*). *Lontra felina *recently diverged from *L. provocax*, possibly from populations in the SFC that progressively adapted to the coastal marine habitat [37].

The present study analysed the phylogeographic pattern and the population structure of *L. provocax*, based on the mitochondrial DNA sequences from control region (CR), the NADH dehydrogenase subunit 5 (ND5) and the cytochrome b gene (Cyt-b). We aimed to infer: i) the demographic processes associated with the LGM south of the Pleistocene ice cover limit for *L. provocax *populations; ii) the evolutionary relationship between freshwater and marine populations; iii) the population structure within each habitat type, among different continental river basins and across the Andes mountain range, comparing *L. provocax *populations from Chile and Argentina.

## Results

A total of 569 bp from the mitochondrial DNA control region (CR), 575 bp for ND5 and 1,007 bp of the cytochrome b (Cyt-b) were sequenced from a total of 75 samples. A total of 11 CR haplotypes, 6 ND5 haplotypes and 9 Cyt-b haplotypes were found for *Lontra provocax *(Table 1). Although one CR haplotype did not illustrate a clear geographic separation, ND5 and Cyt-b haplotypes showed a clear partition according to different environments. Three ND5 haplotypes (I, II, III) were exclusively from continental freshwater (CRL), whereas haplotype IV was distributed from the Chiloé Island rivers (CI) to the southern fjords and channels (SFC) and Nahuel Huapi area in Argentina. Haplotypes V and VI were exclusively found in SFC (Table 1). On the other hand, the Cyt-b haplotypes (A, B and I) found in both continental and Chiloé Island freshwater habitats (CRL and CI), were not shared with SFC marine habitat, which showed a high diversity of Cyt-b haplotypes (C, D, E, F, G, H; Table 1). The Partition Homogeneity Test (*P *= 0.1767) indicated statistical congruence between the three mtDNA sequences (CR+ND5+Cyt-b), therefore concatenated sequences were used for data analysis. Seventeen haplotypes and 26 polymorphic sites were observed from the concatenated mtDNA sequences.

**Table 1:** Sampling sites of *Lontra provocax *analysed in this study.

Localities code	Group	Locality name	Geographical coordinates		Sample Size	Haplotype
					
			Latitude (S)	Longitude (W)		
**CRL-1**	**Continental**	Huilio River, Chile	38°58'	73°01'	2	A.I.A
**CRL-2**	**rivers and lakes**	Queule River, Chile	39°12'	72°55'	5 (4)	A.I.A; B.II.A
**CRL-3**		Mahuidanche River, Chile	39°13'	72°50'	1	A.I.B
**CRL-4**		Lingue River, Chile	39°27'	73°05'	8	A.I.A
**CRL-5**		Cua cua River, Chile	39°42'	71°54'	3 (1)	A.I.A
**CRL-6**		Riñihue Lake, Chile	39°46'	72°27'	2	A.I.A
**CRL-7**		Traful Lake, Argentina	40°30'	71°35'	1	A.IV.I
**CRL-8**		Nahuel Huapi Lake, Argentina	41°05'	71°35'	4	A.IV.I
**CRL-9**		Petrohue River, Chile	41°08'	72°24'	4	C.III.A
						
**CI-10**	**Chiloé Island**	Darwin, Chiloé Island	41°52'	73°39'	5 (1)	A.IV.A
**CI-11**		Chepu River, Chiloé Island	42°02'	73°58'	4	A.IV.A
						
**SFC-12**	**Southern Fjords**,	Tictoc Island	43°38'	73°01'	2	J.IV.C; D.IV.C
**SFC-13**	**and Channels**	Melinka	43°53'	73°44'	(2)	A.IV.G
**SFC-14**		Queulat River	44°27'	73°35'	9	E.IV.C; A.IV.C
**SFC-15**		Seno Magdalena, Magdalena Island	44°35'	72°56'	9 (1)	A.IV.C
**SFC-16**		Valle Marta, Magdalena Island	44°52'	72°55'	1	A.IV.C
**SFC-17**		Puyuhuapi Channel, Magdalena Island	44°44'	72°50'	4	E.IV.C
**SFC-18**		Puerto Aguirre	45°09'	73°31'	(4)	F.IV.H, A.IV.G
**SFC-19**		Madre de Dios Island	50°00'	75°07'	1	K.V.D
**SFC-20**		Around Puerto Natales	51°43'	72°29'	1 (1)	I.V.D
**SFC-21**		Around Punta Arenas	53°08'	70°54'	3 (3)	D.V.D; H.IV.F; G.VI.E

Sampling sites are divided by habitat type (CRL-continental rivers and lakes, CI- Chiloé Island or SFC-Southern Fjords and Channels) and locality numbers (Figure 1, 2), including geographic coordinates, sample size (in parenthesis included the number of samples obtained from tissue), and concatenated haplotypes found for each location.

The concatenated haplotype A-I-A (for CR-ND5-Cyt-b respectively) was widely distributed in the continental freshwater habitat, except in Petrohue where haplotype C-III-A was observed. Haplotypes A-I-B and B-II-A were also found only at Mahuidanchi River and Queule River respectively. A unique haplotype A-IV-I was found on Nahuel Huapi area in Argentina, not detected on Chilean rivers. All samples from rivers on Chiloé Island had a unique haplotype A-IV-A, which was not found in continental or marine sites. In the SFC, each haplotype was restricted to one or few locations.

The SAMOVA revealed increasing *Φ_CT _*values when the numbers of groups increased (K = 2-6; *Φ_CT _*= 0.55-0.72). Petrohue River was separated out in all SAMOVA partitions. The most geographically coherent partition could be defined in 4 groups: (*i*) most continental rivers and lakes locations including Chiloé Island (location 1 to 11), (*ii*) continental Petrohue river (location 9), (*iii*) fjords, channels and Queulat River (locations 11 to 18) and (*iv*) southern-most fjords and channels (locations 19 to 21). High population structure (*Φ_ST _*= 0.85, p < 0.0001) was found among 21 populations and the four groups defined. No haplotype was shared between the four groups.

We found an overall high haplotype diversity (0.8775 +/- 0.0195) but low nucleotide diversity (0.001610 +/- 0.000922) for *L. provocax*. The expected pattern of a lower diversity for the glaciated region was not observed, since the haplotype diversity of the southern group (*h *= 0.7873 +/- 0.0506 Table 2) was high and comparable to that of the non-glaciated area, such as the continental freshwater habitat (*h *= 0.6092 +/- 0.0869, Table 2) or the entire northern region (Chiloé Island + continental rivers and lakes in Chile and Argentina, *h *= 0.7220 +/- 0.0537).

**Table 2:** Genetic diversity of mtDNA sequences for *Lontra provocax*.

Geographic areas		RC		ND5		Cyt-b		RC+ND5+Cyt-b - Genetic Diversity			
	*N*	S	Hap	S	Hap	S	Hap	S	Hap	*h*	π
Continental rivers and lakes	30	5	3	4	4	2	3	11	5	0.6092+/- 0.0869	0.001306 +/- 0.000791
Chiloé Island	9	0	1	0	1	0	1	0	1	0	0
Southern Fjords and Channels	36	8	9	2	3	6	6	16	11	0.7220 +/- 0.0531	0.001060 +/- 0.000663
											

**Total**	**75**	**12**	**11**	**6**	**6**	**8**	**9**	**26**	**17**	**0.8775 +/- 0.0195**	**0.001610 +/- 0.000922**

Sample size, polymorphic sites (S), number of haplotypes (Hap) for each of mtDNA sequences (CR, ND5 and Cyt-b), haplotype diversity (*h*) and nucleotide diversity (π) for the concatenated sequences CR+ND5+Cyt-b by habitat.

**Figure 3:**
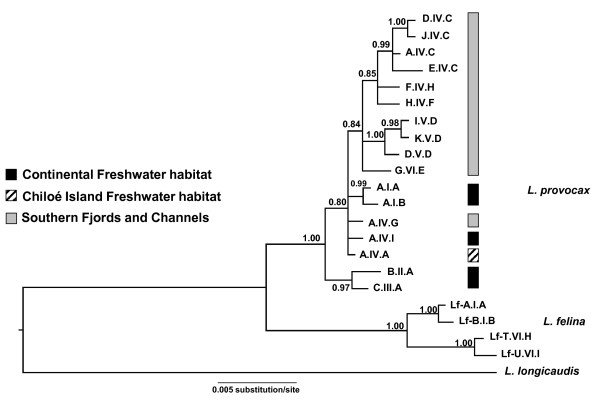
**Bayesian phylogenetic tree constructed using 2151 bp of mitochondrial DNA CR, ND5 and Cyt-b concatenated haplotypes**. Four *L. felina *haplotyes were incorporated on the phylogeny, as well *L. longicaudis *as outgroup. Nodes support values are presented as Bayesian posterior probabilities. Black colours represent haplotypes from continental freshwater habitat, diagonal black lines represent rivers from Chiloé Island and gray colour is haplotypes from Southern Fjords and Channels.

The model selected for the three concatenated genes was TrN+I, selected based on the lower value of Akaike information criterion (AIC). The Bayesian phylogenetic analysis (BA; Figure 3) showed two divergent clades including haplotypes described for continental freshwater habitat, whereas haplotypes from Chiloé Island (A.IV.A) and Nahuel Huapi (A.IV.I) belonged to a polytomous group of mixed origins. A continental freshwater clade is represented by two haplotypes widely distributed along continental rivers and lakes (A.I.A and A.I.B), and another one by two haplotypes (B.II.A and C.III.A) found on Queule (39°12'S) and Petrohue rivers (41°08'S). Another clade was represented by the majority of haplotypes from the SFC. One of the SFC haplotypes (A.IV.G) was found within the paraphyletic assemblage consisting of freshwater haplotypes.

The divergence between *L. felina *and *L. provocax *was 1.5%, whereas it reached 5.2% between *L. felina *and *L. longicaudis*, and 4.6% between *L. longicaudis *and *L. provocax*. The mean distance within the *L. provocax *clade was 0.3%. *L. provocax *mean distance between clades varied from 0.5% between a freshwater clade (B.II.A+C.III.A) and the SFC clade, 0.4% among continental freshwater clades (A.I.A.+A.I.B and B.II.A.+CI.II.A), to 0.1% among the freshwater lineages (A.I.A.+A.I.B, A.IV.A, A.IV.I, A.IV.G).

**Figure 4:**
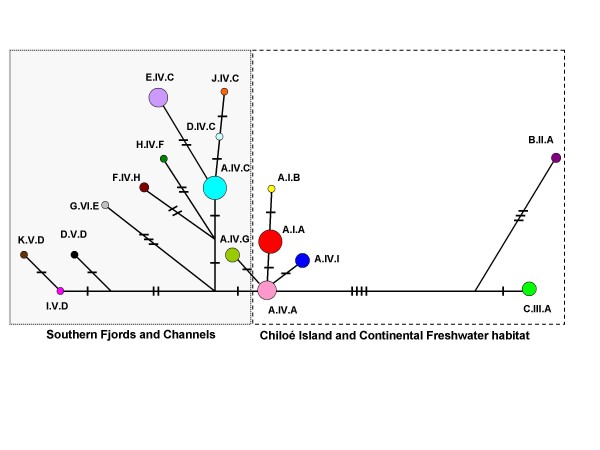
**Median Joining Network of CR+ND5+Cyt-b haplotypes**. Coloured circles represent haplotypes such as the figure 1 and 2. Haplotypes from the different environments Southern Fjords and Channels, Chiloé Island and Continental Freshwater habitat are indicated.

**Figure 5:**
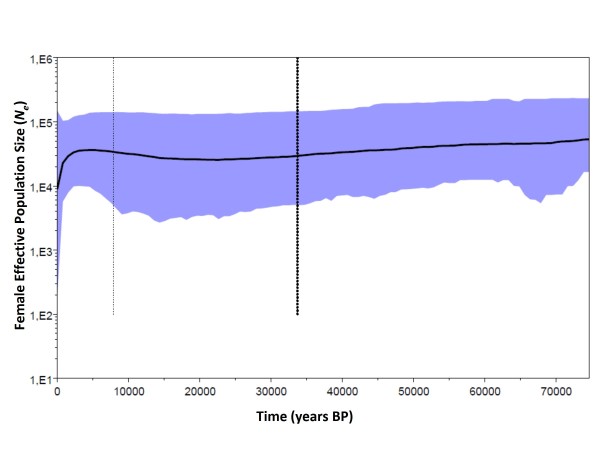
**Bayesian Skyline plot of populations from areas covered by ice sheet during LGM (clade from SFC) showing the effective population size stability throughout time**. X axis: time in years BP, Y axis: is *N_e _*(female effective population size). The middle line is the median estimate, and the grey area shows the 95% highest probability density (HPD).

A signature of recent expansion was detected from the SFC clade, as evidenced by the significant Fu's test (*Fs*= -3.57, *P *= 0.03), although Tajima was not significant (*D*= -1.12, *P *= 0.13). The observed mismatch distribution was not significantly different from that expected for both models, however the spatial expansion model fit the SFC-clade data better than demographic expansion model (SSD *P*-value = 0.80, SSD = 0.02 for spatial expansion vs. SSD *P*-value < 0.001, SSD = 0.30 for demographic expansion). In contrast, the Median Joining Network (MJN; Figure 4) topology for the entire distribution of the SFC haplotypes, which differs from that expected in a recent population expansion (*i.e. starlike *phylogeny with retention of the ancestral haplotype). MJN indicates high divergence among haplotypes from freshwater habitat and a lack of intermediate haplotypes. Moreover, the Bayesian skyline plot did not show any evidence of historical population growth for the populations from the SFC clade, but rather a recent population decrease (Figure 5). No signature of recent expansion was detected for *L. provocax *from the distribution in non-glaciated freshwater regions (*D*= -0.29, *P *= 0.43; *Fs *= 1.34, *P *= 0.78).

## Discussion

Our data evidenced a strong genetic differentiation between continental freshwater and SFC regions. The limits of the latter correspond not only to a habitat change, but also to a major biogeographic break for marine [14] and freshwater [15] species, and to the northern limit of coastal LGM ice cover [2,3].

### Southern marine fjords and channels: glacial survival?

Population displacements followed by founder effects due to recolonization of southern areas from a northern refuge would produce a signature of reduced genetic diversity, when compared to northern areas. The latter pattern was described for southern bull kelp (*Durvillaea antarctica*), which has reduced genetic diversity in southern Chile with a clear signature of postglacial expansion [38]. In our case, phylogenetic reconstruction for *L. provocax *indicates that most haplotypes from SFC form a distinctive haplogroup emerging from a basal northern haplotype found in freshwater habitat, which could suggest a postglacial recolonization from the northern freshwater refuge. However, several results suggest a different scenario: a non-starlike network of haplotypes from SFC, an absence of historical population growth signature from the skyline plot analysis, high haplotype diversity, and highly divergent lineages. These results are not in agreement with a scenario of post-glacial expansion from northern freshwater populations. They rather support the hypothesis of a persistence of the species in this region during LGM. The hypothesis of persistence in glaciated areas is most often rejected by phylogeographic studies. Some studies have, however, shown phylogeographic results strongly supporting demographic persistence in areas supposedly covered by ice [39].

In Patagonia, iso-pollen lines indicate the existence of a terrestrial southern refuge [4] along the south-eastern coast of the Island of Tierra del Fuego, east from the Beagle Channel. The presence of a southern refuge would explain the present day plant species disjunction between Chiloé and Magallanes, and the presence of Bryophyta species and vascular herbaceous plants not distributed further north of 47-48°S [40]. Nevertheless, the unique southern glacial refugium described for terrestrial species was not supported by our data. Single or multiple southern refugia and the persistence of the species within glaciated areas in Chilean Patagonia were debated for terrestrial [7-9,34,35,41-46] and freshwater species [10-13,47,49]. In the case of temperate forest species, patches persisted either at the northern limits of ice cover in Chile or on the eastern limits of ice sheet in Argentinean Patagonia [34,35,42,44,48]. All these examples point to a post-glacial colonization of western Patagonia, *i.e*. the Chilean side of the Andes. The survival of *L. provocax *in non-glaciated areas of the eastern side of the Andes would have required a recolonization of the species across the Andes at multiple sites in order to allow the re-introduction of such a high genetic diversity. In addition, because the species currently occurs only in the Nahuel Huapi and Limay River area in continental Argentina [24-26], subsequent localised extinctions of *L. provocax *along most of the eastern refugia would be required to support such a scenario. Lastly, the unique Argentinean haplotype (A.IV.I) is derived from haplotype A.IV.A present in Chiloe Island, suggesting that its distribution was likely widespread in the recent past, and that range expansion was more likely from Chile to the eastern side of the Andes. Thus, the results do not support an origin of *L. provocax *diversity from eastern Patagonia.

On the other hand, glacial refugia on western coast of the Andes were suggested for some freshwater species. MtDNA sequences of the fishes *Galaxias platei *and *G. maculatus *along the western side of the Andes mountain (39°-49°S) suggest that these species survived in small southern sites due to discontinuities of the ice field [12,49]. Also, exposed portions of the Pacific continental shelf could have constituted favourable environment for such aquatic species [12]. Similarly, the freshwater crab *Aegla alacalufi *seem to have survived in glaciated areas, at least in a site identified as the El Amarillo hot springs, which was possibly left uncovered by glacial ice [13].

Whether multiple refugia existed or *L. provocax *survived all along the western southern Patagonia during LGM is still a matter of debate. The persistence of *L. provocax *is dependent on terrestrial habitat for dens (shelter) and on aquatic habitat for food availability. Such habitat was probably available on the eastern side of the Andes, as suggested by the extension of the distribution of *Fitzroya cupressoides *[34], allowing the survival of *L. provocax *in a large area and therefore allowing the persistence of high genetic diversity. In the SFC, *L. provocax *is known to feed not only on crustaceans and sea urchins, but also on intertidal and subtidal fishes (46% of its diet) [28]. Ice scour can eliminate intertidal and shallow water benthos in the Southern Ocean [50]. In the case of complete elimination of intertidal resources, diet could have been based on species such as the freshwater fish (*Galaxias platei *and *G. maculatus*), catfish (*Trichomycterus areolatus*) or the freshwater crab (*Aegla alacalufi*), which survived in the area during LGM [12,13,47,49], or subtidal organisms. Otter species, such as North American river otter (*Lontra candensis*), the Eurasian otter (*Lutra lutra*) and the sea otter (*Enhydra lutris*), are distributed throughout extreme cold environments. *L. canadensis *inhabiting the marine environments in Alaska has access to two major types of prey: intertidal-demersal organisms such as fishes (Cottidae, Hexagrammidae) and crustaceans, and seasonally available schooling pelagic fishes [51]. Similarly, *L. provocax *populations could have shifted their diet according to prey availability and thus persisted during the LGM in the Patagonian SFC. Although species such as the Eurasian otter (*Lutra lutra*) show evidence of a unique glacial refuge and low genetic diversity [52], other mustelids were able to survive during LGM. *Gulo gulo, Mustela nivalis *and *Mustela erminea *show adaptations for survival in Pleistocene conditions [53].

### Southern glacial refugia or adaptation to marine habitat

Our data show a monophyletic haplogroup for most haplotypes from the SFC range of *L. provocax *(43°S to 53°S), distinct from freshwater habitat haplotypes. Such differences between the *L. provocax *populations inhabiting the freshwater and marine environments suggest either past genetic isolation, and/or restricted gene flow between them at the present time, allowing genetic drift or natural selection to operate. Changes in the *L. provocax *diet and a greater ability to swim larger distances as required in the SFC could eventually lead to adaptation to the marine environment, if plasticity is limited. However, patterns of genetic diversity generated by gene surfing during recolonization are similar to those generated by selection and could thus be mistakenly interpreted as adaptive events [54]. Similarly to selection and unlike most other demographic effects, gene surfing generally does not affect all loci, and thus seems especially difficult to distinguish from directional selection [55]. All otter species, except the sea otter (*Enhydra lutris*) and the chungungo (*Lontra felina*), are dependent of freshwater habitat [20]. Nevertheless, freshwater sources are abundant in SFC and *L. provocax *can temporarily return to rivers to access a supply of fresh water. Moreover, all but four freshwater otter species have also been recorded along the coast [20] (*Lontra canadensis*, *Lutra lutra *and *Lontra longicaudis*, among others). Nevertheless, no genetic surveys have been conducted to determine divergence of otter lineages from different environments. *Lontra *phylogeny based on mtDNA markers revealed the recent divergence between *L. provocax *and *L. felina *about 883,000 years ago (95% HPD: 0.16-1.89 mya) with a possible speciation of *L. felina *from *L. provocax *living on SFC [37]. This speciation scenario is in agreement with the adaptation hypothesis of *L. provocax *to the marine habitat in SFC.

### Conservation implications

Although higher haplotype diversity was expected along northern populations due to the persistence of rivers and forests, low haplotype diversity (compared to SFC) but high divergence among haplotypes was observed. Our results are concordant with the hypothesis of a recent loss of genetic diversity in freshwater environments due to hunting and habitat destruction. This is specifically supported by: i) A.I.A haplotype shared among several locations; ii) two highly divergent clades; iii) two divergent haplotypes (A.I.A and B.II.A) in the Queule River. Genetic theory predicts that during population bottlenecks low frequency alleles are lost by genetic drift [56]. Similarly, *L. provocax *intermediate haplotypes are not seen on the MJN, suggesting that they were eliminated within the freshwater range. This pattern is consistent with the history of the *L. provocax *populations in the region. Indeed, *L. provocax *populations have been eliminated from the north of its past range. The northern limit of distribution changed from Cauquenes and Cachapoal rivers (34°S) to Tolten River basin (39°S) [21]. Its small geographical range has been strongly impacted by anthropogenic activities resulting in a decline to less than 10% of its former distribution in freshwater habitats. *L. provocax *was intensively hunted for its fur; and hunting continued until the 1970's in some southern localities [21]. Furthermore, the species activity has been significantly reduced in areas where riparian vegetation was removed or watercourses were disturbed or recently polluted by pulp factories [57,58]. Riparian vegetation significantly influences the presence of crustaceans and consequently the occurrence of *L. provocax *in the area [30,32,57].

The reduction in the distribution of *L. provocax *led to the classification of the species in Chile as ''endangered'' in the northern range between the O'Higgins and Los Lagos regions, in rivers and lakes, and ''insufficiently known'' for the Aysén and Magallanes regions, where distribution was largely marine [59]. Although *L. provocax *in freshwater habitats mostly occur below 300 m altitude [33], the majority of National Parks and Reserves (> 90%) in south-central Chile (35.6° to 41.3°S) are located above 600 m altitude [48], and therefore do not serve the conservation of this species.

Our data shows that the survival of the species along SFC during glacial cycles maintained a high diversity along SFC. Large-scale temperate deforestation in Chile has progressed from north to south [60]. Human populations are concentrated in the central-south region of Chile, and are less dense south of Chiloé Island. Thus, southern *L. provocax *populations have not been greatly impacted by anthropogenic actions, and have maintained high genetic diversity compared to northern freshwater populations. *L. provocax *populations along SFC are, however, barely studied, and increasing human activities in this area are a potential threat to these populations.

## Conclusions

Our results evidenced the persistence of a semi-aquatic carnivore species, the huillín, in western Patagonia along areas covered by ice sheet during LGM. Marine habitat of the SFC played an important role for *L. provocax *survival during LGM, probably associated to the survival of other freshwater and marine species that may have represented a persistent food source for the huillín. Therefore, genetic differentiation between northern exclusively freshwater habitat dominated by riparian vegetation and SFC may be explained by some ecological differentiation between both kinds of habitats. This is an interesting clue for understanding why so many aquatic species seem to have persisted in glaciated areas.

## Methods

### Study area, sample collection and DNA extraction

A total of 57 feces and 18 tissue samples were collected. These included blood from captured animals, muscle from carcasses of animals that died of natural causes, and pelts confiscated by authorities due to illegal hunting. Feces and muscle tissue were preserved in pure ethanol. The sampling range included the full range of the species (Figure 1) from the northern limit in Chile, the Huilio River tributary of Tolten river (38°58'S) to southern Patagonia (53°08'S, Table 1, Figure 1, 2). In addition we sample the only known population of the eastern side of the Andes: the Nahuel Huapi Area. A total of 21 locations were sampled, nine in continental rivers and lakes (CRL), two in rivers from Chiloé Island (CI) and ten marine locations in the fjords and channels (SFC).

### PCR amplification

Mitochondrial DNA control region (CR), NADH dehydrogenase subunit 5 (ND5) gen and cythocrome b (Cyt-b) gen were amplified using primers described by [37]: LfCR-2F and LfCR-1R; ND5-DF1 and LfND5-R; and LfCYTB-1F; LfCYTB-1F; LfCYTB-2F and LfCYTB-2R.

PCR reactions were carried out following [37]. PCR amplification was confirmed by electrophoresis of products with ethidium bromide in 0.8% agarose gels and visualization under UV light. Amplicons were purified using QIAquick PCR purification kit (Qiagen) and sequenced using amplification primers by Macrogen Inc., Seoul, Korea. All sequences have been deposited in the GenBank [GenBank: GQ843803-GQ843824, and HM997011-HM997017].

### Data analysis

#### Population analysis

The sequences were aligned and mutations were confirmed by eye according to the chromatogram using Proseq ver. 2.91 [61]. All sequences were aligned and haplotypes were identified using ClustalX ver. 1.83 [62].

Spatial analysis of molecular variance (SAMOVA) was implemented by SAMOVA ver. 1.0 [63] to define groups based on the geographic distribution of the genetic diversity. SAMOVA were performed for 21 locations testing from 2 to 7 groups, each of which with 100 initial conditions. The groups of populations geographically homogeneous are defined by maximizing *Φ_CT _*values (among group variance) and minimizing *Φ_SC _*values (among populations within group variance). Haplotype (*h*) and nucleotide diversity (π) were calculated using Arlequin program ver. 3.0 [64] for all data set and for the different environments.

Deviations from a neutral Wright-Fisher model were performed by calculating Tajima's *D *and Fu's *Fs *statistics [65,66]. We tested the demographic [67] and spatial expansion [68] models by calculating the sum of squared differences (SSD) between the observed and an estimated mismatch distribution obtained by 1,000 bootstrap. The *P*-value of the SSD statistic was calculated as the proportion of simulated cases that show a SSD value distinctive from the original. Calculations were performed in ARLEQUIN, using 1,000 bootstrap to evaluate significance. To estimate the shape of population growth through time for the individuals distributed along the ice sheet coverage area during the LGM, we constructed Bayesian skyline plots implemented in BEAST v 1.4.8 [69]. The appropriate model of nucleotide substitution was HKY+I determined using ModelTest ver. 3.06 [70]. Five million iterations were performed, of which the model parameters were sampled every 1000 iterations. Throughout our analysis, we assumed a within-lineage per site mutation rate of 6%Ma. Demographic plots for each analysis were visualized using Tracer v1.0.1 [69].

#### Phylogenetic analysis

We applied the Partition Homogeineity test (10,000 permutation) to assess the congruence of the evolution rates among CR, ND5 and Cytb using PAUP ver. 4.0b8 [71]. The evolutionary relationship among concatenated CR+ND5+Cyt-b haplotypes was investigated by a Median Joining Network using Network ver. 4.5.1.0 [72], and phylogenetic reconstructions based on Bayesian (BA) methods. Four divergent haplotypes of *Lontra felina *[37], were incorporated in the phylogenetic reconstruction, whereas a *L. longicaudis *haplotype from the Amazon (this manuscript) was used as an outgroup. The substitution model of DNA evolution was selected based on AIC using Modeltest ver. 3.06 [69]. BA was performed by MrBayes ver. 3.1.2 [73] using the general type of the best fit model parameters defined for the data set, in which four independent analyses were run with four chains each, for six million generations and then sampled at intervals of 1,000 generations. The first 25% of sampled trees were discarded to ensure stabilization and the remaining used to compute a consensus tree. The split frequency was below 0.004, confirming that sampling was from the posterior probability distribution. Mean distance between clades and species was calculated using Mega v.3.1 [74] using p-distance.

## Authors' contributions

JAV conceived of the study, participated in sample collection, molecular genetic studies and in statistical analyses and drafted the manuscript. GMV participated in the design of the study and helped to draft the manuscript. CC helped on the field work and results interpretation based on his experience on the species to draft the manuscript. WS helped on the field work and results interpretation based on his experience on the species to draft the manuscript. CO helped on the field work and to draft the manuscript. SF participated in the design of the molecular genetic studies, contributed to the discussion of results and to the interpretation and drafted the manuscript. All authors read and approved the final manuscript.
